# Supplementary material for: Are carbon emissions trading and green financial instruments synergistic? -Comprehensive quantitative research based on content analysis
Source: PLoS One. 2024 Mar 7;19(3):e0298601. doi: 10.1371/journal.pone.0298601 (PMC10919601; doi:10.1371/journal.pone.0298601)
Supplement: S1 Data — (ZIP) [file pone.0298601.s001.zip › Data/地级市Z_F工作报告.zip!/║╙▒▒/╒┼╝╥┐┌╩╨/└┤╫╘╣1⁄2╓┌║┼í╛┬φ┐╦╩2╛▌═°í┐_2019.docx]

各位代表：

现在，我代表市人民政府，向大会报告工作，请予审议，并请各位市政协委员和列席人员提出意见。

一、2018年工作回顾

过去的一年，在习近平新时代中国特色社会主义思想指引下，我们坚决贯彻习近平总书记视察张家口重要指示精神，全面落实党中央、国务院和省委、省政府决策部署，在市委坚强领导下，按照“三六八九”工作思路，讲学习、转作风、抓落实，协调推进稳增长、促改革、调结构、惠民生、防风险各项工作，努力践行新发展理念，深化供给侧结构性改革，紧紧围绕“冬奥会筹办、脱贫攻坚、建成首都水源涵养功能区和生态环境支撑区”三大历史任务，锐意改革、勇于担当，拼搏竞进、扎实苦干，经济社会各项事业取得了新成就，较好完成了市十四届人大三次会议确定的目标任务。

———经济发展稳中有进，总体向好。全市生产总值增长7.6%，全部财政收入增长18.5%、首次突破300亿元， 一般公共预算收入增长15.5%，固定资产投资增长7.4%，规模以上工业增加值增长13.8%，社会消费品零售总额增长8.9%，实际利用外资增长7.3%。预计城乡居民人均可支配收入分别增长9%和11％以上，万元生产总值能耗下降4%左右。主要经济指标增速均位于全省前列。

———冬奥会筹办工作高质量有序高效推进。对标“精彩、非凡、卓越”目标，贯彻“四个办奥”“五个着力”要求，扎扎实实推进各项筹办工作。高水平完成了“10+7”规划体系编制和重点项目设计，76个场馆和相关配套基础设施项目开工了65个。云顶滑雪公园、“三场一村”、太子城冰雪小镇、张家口南综合枢纽、宁远机场和住宿保障等重点项目快速推进。完成冬奥核心区场馆和市政统筹规划，成立冬奥核心区工程建设指挥部，组建工程质量和施工安全监管工作专班，全面加强工程质量监管。住宿、餐饮、医疗、安保、交通、志愿者6个领域保障方案初步编制完成。编制了冰雪运动和冰雪产业发展规划，积极发展冰雪运动和冰雪产业，承办国际国内体育赛事70项，举办群众性冰雪运动100项，建立冰雪运动特色学校62所。积极推动冰雪运动全覆盖，发展零度以下经济，“红红火火中国年”系列活动在全市普遍开展，参与冰雪运动人数达274万人次，增长112.3%。着力深化国际交往，加强国际合作和工作交流，与芬兰拉赫蒂市、法国尚贝里市缔结友好城市。签约冰雪运动装备项目21个，注册企业18家，法国MND索道等3个项目已经投产。

———脱贫攻坚扎实深入，成效显著。全市共投入各级各类财政扶贫资金42.78亿元，增长45.07%。认真抓好国家、省扶贫考核反馈意见整改，深入开展扶贫领域腐败和作风问题专项治理。协调发挥中直帮扶、北京对口帮扶、省“五包一”、工作专班、驻村工作队各方作用，形成了强大工作合力。出台了《关于打好2018年精准脱贫攻坚战的实施意见》《张家口市脱贫攻坚三年行动计划（2018-2020年）》，扎实推进“六个精准”“五个一批”，着力构建专项扶贫、行业扶贫、社会扶贫“三位一体”扶贫大格局。实施产业扶贫项目3210个， 带动贫困人口27.84万人，100.54万千瓦光伏扶贫电站并网运行， 助力8.26万户贫困群众稳定脱贫。119个易地搬迁集中安置项目已交付94个，搬迁入住5.2万人。组织2.2万贫困人口参与生态建设，为贫困人口提供生态护林员、河湖巡查员公益岗位3.95万个。对4.5万名贫困家庭学生进行教育资助，22.9万贫困对象纳入农村低保和特困供养保障范围，对所有建档立卡贫困人口医保个人缴费给予全额资助。大力改善基础设施和公共服务，完成农村危房改造3.12万户，解决13.4万农村贫困人口饮水安全问题，新建、改建“四好农村路”2123公里，全市行政村基本实现硬化路、通班车，新建、改建村卫生室253个，实现贫困人口家庭医生签约服务全覆盖。加强社会扶贫工作，建立京张对口帮扶和扶贫协作工作管理机制，总投资20.26亿元的183个扶贫协作项目全部开工，完工127个。积极推进“互联网+扶贫”模式，在“中国社会扶贫网”注册爱心人士24.8万人、贫困户6.5万户，物品需求对接成功超过3000件，资金需求对接成功达到300万元，发展势头比较好。大力推进“四方联动”扶贫开发新机制，通过扶农公司流转土地37万亩、 引进市场主体178家。全市17.8万贫困人口实现稳定脱贫，994个贫困村出列，崇礼、宣化、万全、涿鹿4个贫困县区可望脱贫摘帽。

———立足“两区”建设，生态环境建设又上新台阶。扎实做好中央环保督察“回头看”问题整改，持续开展“蓝天、碧水、绿地、净土”行动，完成植树造林601万亩，占全省任务的五分之三，创造了造林绿化的空前奇迹，书写了张家口绿色传奇。全市林木绿化率达到50%。启动13个草原公园和示范牧场试点，创建农业公园试点11个。获批设立“怀来国家森林公园”，全市国家森林公园达到4处。治理水土流失面积215平方公里，压采地下水800万立方米。整治“散乱污”企业1292家，淘汰燃煤锅炉1663台，退出矿山105处。淘汰老旧机动车1.74万辆，推广新能源车4518辆。 新增清洁能源供暖面积335.5万平方米，成功入选北方地区冬季清洁取暖试点城市。497家建筑工地全部安装PM10在线监测设备。空气质量综合指数4.11，PM2.5平均浓度29微克/立方米，继续保持京津冀地区最好水平。“两区”建设规划已完成报审稿。

———坚持创新驱动，加快转型升级，绿色产业体系初步形成。扎实推进系列三年行动计划，坚决去、主动调、加快转。压减煤炭产能860万吨，超额完成省下达任务。加快培育发展新兴产业，冰雪、旅游、大数据、高端制造、可再生能源、食品加工产业发展势头良好。高新技术产业增加值增长34.5%，战略性新兴产业增加值增长3.9%，服务业占比进一步提升，三次产业结构由14.1∶35.8∶50.1调整为14.8∶33.7∶51.5。吉利领克汽车正式量产，沃尔沃发动机工厂、吉利领克工厂被工信部评定为“两化融合管理体系贯标试点企业”。“北方硅谷”集聚效应进一步凸显，入驻企业32家，智慧互通爱泊车的智慧停车项目被工信部列为“2018年人工智能与实体经济深度融合创新项目”。签约大数据项目22个，新投运服务器14万台，累计达到22万台，居全国前列，张北县获评“全国首批大数据产业国家新型工业化产业示范基地”。全域旅游加快推进，新建旅游重点项目11个，新批复国家4A级景区2家，蔚县获批国家历史文化名城。成功举办第二届旅游发展大会，推出桑洋河谷旅游新品牌。新增规模以上工业企业50家，20家经济开发区入驻企业1359家，16家省级经济开发区实现主营业务收入2442.2亿元。科技创新步入快车道，145家高新技术企业进入省后备培育库，新增省市科技创新平台125家，新增高新技术企业68家、认定科技型中小企业405家。新建院士工作站6家。与中科院遥感所合作的亚洲领先的合成孔径微波雷达实验场建成运营。

———发储输用并举，可再生能源示范区建设实现创新突破。新增可再生能源装机174.3万千瓦，总装机达到1345.5万千瓦，位居全国前列。世界规模最大的±500千伏多端柔性直流示范工程加快建设，张北至雄安新区1000千伏特高压获得核准。100兆瓦压缩空气储能、可脱网运行的黄帝城小镇多能互补示范项目列入中科院先导A专项。深入推进“四方协作”机制，完成市场化交易电量3.75亿千瓦时，有力促进了清洁供暖，经验做法得到国务院办公厅通报表扬。可再生能源消费量占终端能源消费比例达到23%，居全国领先水平。与德国欧能、太阳贝等5家欧洲企业签署合作协议，龙驰锂电池等20个装备制造项目开工建设，金风科技总装智能工厂正式投产。编制了氢能城市规划，亿华通氢燃料电池发动机投产，76辆氢燃料公交车投用，制氢、加氢设施建设加快，氢能产业全链条发展态势已经形成。

———注重基础先行，城镇化进一步加快。加快推进国土空间规划编制，落实“五级两规一导则”体系，加强城乡规划统筹，加快城乡基础设施建设，促进城乡一体化发展。城建重点项目完成投资346.9亿元，投资完成率创历史新高。“未来之城”规划建设全面启动，洋河新区、奥体中心、张家口南综合枢纽等节点区域开发建设加快。城市“双修”试点工作有序推进，虹桥北路、中兴北路地下管廊示范工程启动建设，完成“海绵城市”道路、广场示范工程5处，改造各类老旧市政管网158公里，主城区新建改造城市道路29条，新增绿地135万平方米，我市顺利通过国家园林城市复查，清水河水利风景区晋级国家水利风景区。入选省优先发展公共交通示范创建城市，公交首期大站快线投入运行。交通一体化和立体化进程加快，机场新航站楼地下基础工程已完成，京张高铁、崇礼铁路加快建设，太行山高速建成通车，二秦、京新三期高速具备通车条件，延崇高速顺利推进。阳原县成功创建省级园林县城，怀安县通过省级园林县城复查。中心城区和城镇承载能力进一步增强，全市常住人口城镇化率达到57.5%。

———注重改革创新，发展活力和内生动力进一步增强。大力推进“放管服”改革，全面实施“五十证合一、一照一码”，投资项目审批时间由180天缩减到30天，企业开办时间由20天缩减到5天。加快建设“网上政府、身边政府”，在全省率先实现全部市级政务服务事项网上可办，建成239个便民服务中心、4254个便民服务室，为企业和基层群众提供全程帮办代办服务。市长热线和群众服务热线办结率达到95%以上，我市成为全省唯一入选12345热线全国 “最佳进步榜”城市。深入推进国企改革，完成9家市级经营性国有资产集中统一监管、6家全民所有制企业公司制改制和18家国企办社会职能的剥离工作。深化投融资改革，新增挂牌上市企业7家、设立基金5支，中信银行分行、张家口农村商业银行正式营业。创建国家全域旅游示范区等9项改革全国领先，4项改革经验在全省推广。深入推进农业供给侧结构性改革，实施乡村振兴战略，制定了规划和实施意见，积极有序推进农村承包地和宅基地两个“三权分置”改革，大力实施农业龙头企业提质扩模带行业行动，开展农村人居环境整治三年行动，有效助推了脱贫攻坚和乡村振兴。认定省级农业科技园区17家、特色农产品优势区10个，新增14个省级名牌产品和 “张北马铃薯”“南口供佛杏”2个农产品地理标志产品，“三品一标”动态认证面积达到148.11万亩，有机动态认证面积达到6.4万亩。4174个行政村全部实现生活垃圾有效处理，启动了117个垃圾污水治理、农村“厕所革命”、“四好农村路”建设、燃煤置换“四到位”示范村镇建设。

———坚持以人民为中心，民生保障水平进一步提升。加大民生事业投入，全年民生领域支出占公共预算支出的81%，增长18.9%。教育事业加快发展，新建成24所普惠制幼儿园，全市学前教育毛入园率达到85.22%；新建改建义务教育薄弱学校校舍3万平方米，通过国家义务教育发展基本均衡县评估认定县区新增6个。就业再就业成效明显，城镇新增就业8万人，城镇登记失业率3.1%。在全省率先建立“两中心两站两基金”服务管理体系，为1.25万名存在政策欠账的退役军人全部落实了政策，推荐再就业岗位9615个， 安置符合条件的退役士兵277名。医疗保障能力显著增强，实现27个省市异地就医直接结算，19个县区3325家卫生室开通医保刷卡系统。市第一医院晋升为三级甲等医院。北医三院崇礼院区正式揭牌运行。社会保障体系更加健全，新建改建社区和居家养老服务机构10所、农村幸福院78所，城乡低保标准分别提高到7200元和4000元。全力保障群众基本住房需求，完成老旧小区改造项目56个，新开工棚户区改造2.9万套。农村改厕13.99万座。积极组建物业管理集团，接收无物业小区240个。发放住房公积金贷款155.93亿元，帮助8.45万个家庭改善了住房条件。文化事业加快发展，张家口工业文化主题公园完成主体工程，6个县区启动“两馆一中心”项目。顺利完成农村“两委”换届工作。查办食品药品安全领域违法案件1251起，全市80%以上用餐配送单位量化等级达到良好以上。深入开展“扫黑除恶”专项斗争，打掉黑恶势力团伙56个， 破获刑事案件272起，抓获犯罪嫌疑人394人。

一年来，我们大力推进政府自身建设，坚决落实全面从严治党要求，认真做好中央、省巡视反馈意见整改，严格落实中央八项规定及实施细则精神，实施督办、督查和监察递进督促问责机制，持续改进作风，政府公信力和执行力不断提升。大力加强法治政府建设，认真贯彻落实《宪法》，自觉接受人大法律监督、政协民主监督和社会监督，办理人大代表建议262件、政协委员提案432件，办结率100%。全力推进“双创双服”，成立“工业企业事务帮办中心”，为企业减税降费37.89亿元。帮助394家包联企业解决问题868个，问题解决率达到93%。民营经济发展活力进一步增强，净增市场主体3.05万户、企业9341家。

一年来，国防动员、双拥共建和优抚安置工作进一步加强，民族宗教、新闻广电、外事侨务、人民防空、史志档案、气象地震、地理信息、援疆援藏、妇女儿童、老龄、残疾人等事业都有了新发展。

各位代表！过去的一年，我们肩负重大使命，满怀忧患意识，努力抢抓机遇，直面困难挑战，夜以继日、扎实工作，团结奋斗、众志成城。成绩来之不易。这些成绩的取得，是习近平新时代中国特色社会主义思想领航定向的结果，是省委、省政府正确领导的结果，是在市委的坚强领导下、带领全市上下共同努力的结果。在此，我代表市政府，向夙兴夜寐、辛苦工作在筹办冬奥会、脱贫攻坚一线和经济社会发展各条战线的同志们，向全市人民，向人大代表、政协委员，向各民主党派、工商联、无党派人士和人民团体，向驻张解放军、武警官兵和政法干警，向中央、省有关部门，向各界朋友，致以崇高的敬意！表示衷心的感谢!

习近平总书记要求我们要努力交出冬奥会筹办和本地发展两份优异的答卷。我们要不辜负总书记的亲切关怀和厚爱，就必须时刻保持清醒头脑，正视当前存在的问题、风险和困难。一是发展不平衡不充分问题依然突出。我市经济总量还不大，市场主体还不够多，民营经济发展还不够好。二是实体经济困难较多，融资难、融资贵问题仍然比较普遍。三是新旧动能转换还不够快。人才引进数量少，科技创新能力不足，新兴产业规模还不大，还没有形成足够支撑，传统产业改造升级任务艰巨。四是民生领域仍有不少短板。教育、医疗发展水平相对较低。稳定脱贫防返贫任务还比较重。农村人居环境比较差、“空心村”多，治理任务艰巨。五是社会治理和行业监管存在风险隐患。中国化工集团河北盛华化工公司“11·28”重大爆燃事故教训深刻。安全生产、环境保护、社会稳定等领域还存在风险隐患。六是发展环境仍需改善。公共服务需要加快提升，政务服务能力、质量和效率有待提升，营商环境还需进一步优化。一些政府部门和工作人员思想、能力、作风不适应新时代要求，不作为、乱作为消极腐败现象还不同程度存在。我们必须坚持问题导向，以革故鼎新的勇气，采取有力措施，努力加以解决。

二、2019年目标要求和重点任务

今年是新中国成立70周年，是全面建成小康社会关键之年，是我市冬奥会筹办、脱贫攻坚决战决胜关键之年，也是“两区”建设全面推进关键之年。我们必须进一步深入学习领会习近平总书记重要指示精神，增强责任感、紧迫感，只争朝夕，抢抓机遇，创新担当，顽强奋斗。

今年政府工作的总体要求是：坚持以习近平新时代中国特色社会主义思想为指导，全面贯彻党的十九大精神，深入贯彻习近平总书记对张家口工作重要指示批示和中央经济工作会议精神，统筹推进“五位一体”总体布局，协调推进“四个全面”战略布局，全面落实省委、省政府工作部署，在市委的坚强领导下，坚持新发展理念，坚持高质量发展，坚持供给侧结构性改革，坚持改革开放，深入开展“双创双服”活动，抓住重大机遇，扭住工作重点，强化工作统筹，确保重点工作实现突破性进展，为扎扎实实完成三大历史任务、交出冬奥会筹办和本地发展两份优异答卷而不懈奋斗。

今年经济社会发展的主要预期目标是：全市生产总值增长7%， 一般公共预算收入增长10%，规模以上工业增加值增长6%，固定资产投资增长7%，社会消费品零售总额增长9.5%，进出口总值增长5%，城乡居民人均可支配收入分别增长8%和10%，万元生产总值能耗下降3%。PM2.5年均浓度保持29微克/立方米，主要污染物减排完成省下达目标任务。居民消费价格指数涨幅控制在3%以内，城镇登记失业率控制在4.5%以内。这些目标的设定综合考虑了我市的基础、机遇、国内外经济环境，是积极可行的。

落实总体要求，实现任务目标，我们必须坚定不移地坚持党对经济工作的领导，坚定不移地扭住发展第一要务，坚定不移地推动高质量发展，坚定不移地推进改革开放，坚定不移地走创新驱动之路，坚定不移地坚持以人民为中心的发展思想。必须坚持以供给侧结构性改革为主线，统筹推进稳增长、促改革、调结构、惠民生、防风险工作，努力实现经济社会持续健康发展。要着力做好以下八个方面工作：

（一）高标准做好冬奥会筹办工作。围绕举办一届“精彩、非凡、卓越”的冬奥会，坚持“四个办奥”“五个着力”，按照“一刻也不能停，一步也不能错，一天也误不起”的要求，严格按照时间节点，扎扎实实推进各项筹办工作。因势发展奥运经济，推动冰雪运动和冰雪产业在张家口落地生根。注重协同发展，与雄安新区一道“两翼腾飞”。

保质保量推进场馆和相关配套基础设施建设。严格把握开工、竣工验收、测试三个关键节点，细化台账，倒排工期，确保云顶滑雪公园、跳台滑雪场、越野滑雪场、冬季两项中心达到测试赛条件。奥运村主体建成。市中心血站、万龙—转枝莲公路等配套基础设施项目加快推进，山地媒体中心、崇礼—太子城公路等剩余11个项目严格按照时间节点开工建设。充分发挥质量、安全监管专班和专家团队作用，强化工程质量和施工安全监管，确保建成优质工程、廉洁工程。大力推进科技冬奥计划，编制和实施一批示范科技项目，将先进节能智能技术融入场馆建设和运营，助力智慧安保、智慧观赛、智慧交通、智慧旅游。侯卫东官场笔记

科学精细推进赛事服务保障。深化细化6个业务领域保障方案，推进实战化、场馆化、规范化。实施全市住宿业、餐饮业三年提升计划，加快推进各类酒店新建改造、评星评级、服务提升。扎实开展钻石酒家创建和“崇礼菜单”研发，做好冬奥食材基地和供应商备选企业培育推荐工作。适时启动P+R换乘场站规划建设。加快建设由雪场急救站、医疗站、定点医院构成的应急救援链条，抓好医务人员英语、滑雪和救援能力培训。适时组织开展安保多警种区域联防联控实战演练，组织好城市志愿者招募和培训。开展国家跳台滑雪中心、越野滑雪中心、冬季两项中心竞赛场馆运行计划及外围保障计划编制。按照“相约北京”系列赛事要求，组建我市赛区主要测试赛场馆前期工作团队，抓紧筹备2021年自由式滑雪和单板滑雪世锦赛。加强与俄罗斯索契、韩国平昌等冬奥会举办城市和冰雪运动强国交流合作，提升我市开放办奥水平。

大力推广普及冰雪运动。组织承办国际雪联9项世界级冰雪运动赛事，积累办赛经验。扎实开展“红红火火中国年”、冰雪运动全覆盖、第二届冰雪文化艺术节等群众性活动，在中心城区和具备条件的县区建设一批冰雪运动场所，每个县区至少开展1项冰雪主题活动。实施冰雪惠民政策，资助青少年参与冰雪运动，打造一批冰雪运动特色学校，开展冰雪运动学历教育，建设冰雪运动综合培训基地。开展冬奥倒计时1000天主题活动，多形式宣传冬奥，讲好“张家口故事”，营造浓厚奥运氛围。大力发展零度以下经济，加快推进京张体育文化旅游带发展。

加快发展冰雪产业。加强与国内外优势企业合作，大力发展冰雪运动装备研发制造销售、运动旅游、教育培训、赛事服务等冰雪全产业链，打造国家冰雪运动装备生产基地。落实支持冰雪运动装备产业发展若干措施，高新区和宣化区冰雪产业园新注册的18个项目开工建设，实现投产6家以上。加大精准招商力度，年内再新签约项目30个，落地20家，初步形成产业集群。

大力推动协同发展。发挥冬奥会筹办和“两区”建设叠加效应，加强与北京中关村、金融街、亦庄开发区和天津滨海新区等重要创新基地合作，在高铁高速沿线、主要干路沿途、洋河新区和环京三县重要节点区域，加快建设一批高铁新城、特色小镇、专业园区，打造协同发展、协同创新的平台。加强京津冀协同发展项目库建设，精准对接非首都功能疏解和产业转移。大力引进优质医疗教育资源，发挥好北医三院设立崇礼院区的示范带动作用，多形式促进医疗教育机构合作，加快我市医疗教育水平提升。中公教育国际学校、河北工业大学城市学院、北京理工大学怀来校区等项目开工。推动职业学校与京津冀知名学校合作项目达到100个以上，与世界500强企业合作项目达到20个以上。

（二）高质量推进脱贫攻坚。下足绣花功夫，扎实推进脱贫攻坚三年行动计划，聚焦 “两不愁、三保障”，着力在精准和扎实上下功夫，精准扶贫和防止返贫两手抓，脱贫攻坚与乡村振兴有效衔接，确保2019年9.29万贫困人口退出、747个贫困村出列、8个贫困县和赵家蓬区脱贫摘帽。

进一步完善精准扶贫、精准脱贫工作体系。突出“两不愁、三保障、三落实”。统筹推进专项扶贫、行业扶贫、社会扶贫。一方面在精准扶贫、精准脱贫和防止返贫工作无缝衔接上再下功夫，农村全人口台账和“五个一批”台账要动态调整，精准对接，措施及时跟进，做到无漏评、无错评、无错退。大力推进易地扶贫搬迁社区和产业园区“两区同建”，年内实现产业园区配套建设全覆盖。另一方面，统筹考虑“后三年”和“三年后”，在全面梳理“五个一批”联结贫困村、贫困人口，坚持问题导向、分析潜在的漏洞和短板的同时，充分整合帮扶资源，再谋划一批脱贫攻坚与生态建设、城镇化、绿色产业发展有机结合的精准扶贫项目。注重增加村集体收入，注重有劳动能力贫困人口通过产业、就业扶贫项目稳定脱贫全覆盖。

持续改善贫困地区人居环境。扎实推进基础设施和基本公共服务“双基”提升行动。加快1200公里农村公路建设，实施行政村穿村公路硬化工程。推进安全饮水工程，彻底解决贫困村饮水安全问题。持续做好“四类”重点对象存量危房改造工作。加大对非贫困村“双基”提升支持力度。

进一步健全脱贫攻坚长效机制。深入实施“四方联动”扶贫开发新机制，用好农村承包地、宅基地“三权分置”政策，充分发挥扶农公司在推动精准扶贫、绿色发展方面的重要平台作用。结合“空心村”治理，着眼乡村振兴，密切联结脱贫攻坚，加快建设一批广就业、长收益、可持续的生态产业示范项目。沽源老掌沟极限运动小镇一期建成，张北中赫足球小镇开工建设。大力开展精神文明建设，用好孝善基金，在乡村弘扬互帮互助、尊老爱幼、扶困助残的优良风尚。大力褒扬自力更生脱贫致富的先进典型，加强宣传教育，激发贫困群众内生动力。严格督查有关制度的落实，确保扶贫项目资金规范安全。

（三）高水平推进生态建设。立足建成首都水源涵养功能区和生态环境支撑区，抓紧有关工作，争取“两区”建设规划尽快获得国家批准。完善三年行动计划，细化工作方案，扎实实施“蓝天、碧水、绿地、净土”四大行动，坚决打好防治污染攻坚战，建设宜居张家口、首都后花园。

大力推进“蓝天行动”。扎实实施散煤整治、工业污染深度治理等六大攻坚战，实现“散乱污”企业动态清零。严格整治扬尘污染，建筑工地全部做到“六个百分之百”。大力推进“以电替煤”“以电替油”，年内新增清洁能源供暖面积400万平方米。加大新能源汽车推广。加快秸秆综合利用，年内基本全部覆盖。空气质量完成省定目标任务。

大力推进“碧水行动”。进一步深化河长制、湖长制，全面落实河湖“清四乱”工作。实施城市饮用水水源地保护、污染河流治理等专项行动。实施白洋淀流域上游综合整治工程，加快闪电河和官厅水库国家湿地公园建设。改造城镇污水处理设施，县级以上污水处理厂完成除磷脱氮提标和污泥处理设施达标工程。实施压水、节水、兴水、控水、引水相结合，坝上地区实施退耕还草10万亩，退减灌溉20万亩，压采地下水2400万立方米。坝下地区重点支持永定河流域公司加快实施退灌还水工程。

大力推进“绿地行动”。持续推进公司化、林场化造林，完善养护管护机制，巩固好造林绿化成果，进一步科学分析论证，完善坝上坝下绿化造林方案，加快建设林灌草有机结合的复合生态体系。进一步实施沿河、沿路、环城、环村（镇）、环湖和郊野森林公园“两沿三环一公园”造林绿化提升工程，年内完成80万亩。坝上创建草原公园和示范牧场20个，坝下创建农业公园15个。积极推进森林、草原、湿地公益冠名认养和生态资产市场化交易，推动生态资源变资产、资产变资金制度创新。加快矿山退出及生态修复，完成188处责任主体灭失矿山迹地治理任务，退出矿山88处，完成105处退出矿山生态修复工程。

大力推进“净土行动”。实施土壤污染治理与修复规划。完善固体危险物全程监管制度。加强农业面源污染防治。严格禁用0.01毫米及以下厚度地膜，农地膜回收基本全覆盖。积极推广有机肥，发展有机农业，减少化肥农药用量。加快建设垃圾一体化处理体系。

切实强化生态环境监管。深化生态环保管理体制改革，乡镇环保工作机构实现全覆盖。坚决抓好中央、省环保督察反馈问题整改。改进环保监管方式，推进网格化监管体系建设，实现环境监管执法无死角。建立智能监控执法系统，提高生态环境监管执法针对性和时效性。加大惩戒力度，提高环境违法成本。完善重污染应急管理机制，实行一业一策、一厂一策、清单管理，严禁停产限产“一刀切”。广泛宣传发动群众，拓宽环境污染举报渠道，严肃查处环境违法行为，持续提升全市生态文明建设水平。

（四）加快构建现代产业体系。以供给侧结构性改革为主线，坚决贯彻“巩固、增强、提升、畅通”八字方针，坚持创新驱动，着力推动高质量发展。

突出科技创新，加快转型升级。扎实推进质量强市和标准化战略，以高标准引领高质量、以高质量创建名品牌，实现标准、质量、品牌联动提升。深入开展减税降费专项行动，采取切实措施降低实体经济融资成本、制度性交易成本、用人用能成本和拓展市场成本，消除民营企业隐性门槛和不公平待遇。强力推进科技创新、战略性新兴产业发展、工业转型升级、农业供给侧结构性改革、旅游质量提升、电子商务发展等系列三年行动计划。新增高新技术企业60家以上、科技型中小企业300家以上。深入开展“万企转型”行动，利用好中央、省推广技改综合奖补政策，完善我市配套政策，滚动推进100项重点工业项目。大力支持宣钢转型发展。加快工业互联网平台建设和模式应用，加快发展“企业云”和“云上企业”。深入实施军民融合发展战略，加快推进产业聚集，力争军民融合型企业达到30家以上。努力延伸提升完善产业链条，着力发展冰雪、新能源、大数据、高端制造、文化和旅游、特色农牧六大产业集群。市、县（区）两级都要制定切实政策，大力实施县域特色产业高质量振兴计划。

增投资、扩内需，力促经济平稳增长。强化高质量有效投资。开展重点项目建设质量年活动，围绕提高基础设施和公共服务水平大力补短板，在路、水、电、气、讯、生态环保、新型城镇化、教育、医疗等重点领域，协同社会资本、加大投资力度。推进200个市级以上重点项目，完成投资500亿元以上。加快领克汽车、智慧互通、亿华通氢燃料电池发动机、太子城冰雪小镇、腾讯华北信息技术基地、中国电信呼叫中心、教育城等一批重点项目建设。注重发挥政府投资杠杆撬动作用，激发社会资本投资活力，靠大强度有效投资托住全市经济增长的底盘。

大力扩大消费，发挥新需求对经济的拉动力。着力打造体育之城、活力之城、康养之城、文明之城、富强之城，加快发展教育、医疗、康养、文化、会展等现代服务业。加快草原天路全线贯通，办好第三届全市旅发大会，努力创建国家全域旅游示范市。积极发展电子商务，培育发展现代供应链综合服务企业，整合交通运输业资源，促使生产性服务业上档升级。大力支持物业、家政、陪护、月嫂、连锁商贸餐饮、便利店等生活性服务业发展。落实个人所得税专项附加扣除政策，增加居民收入，增强消费能力。提升专业市场，建立生活用品、食品可追溯体系，让人民群众吃得放心、穿得安心、用得舒心。

加快完善协同创新平台体系。完善全市及各县区产业发展规划。落实《关于开发区提升能级打造高质量发展新平台的实施方案》，围绕各园区主导产业定位，精准定制扶持引导政策，着力强链、补链、延链，加快形成产业集聚。加强资源对接，打造一批疏解非首都功能，促进协同发展、协同创新的“微中心”。大力培育“双创”平台。全面推进大众创业、万众创新。大力支持社会资本与政府合作发展大学生创业园、高层次人才创业园、产业孵化器、产业加速器等创新创业平台，在促进就业创业中培育种子企业。积极支持天使基金、创投基金、产业基金、股权基金发展，大力引进各类金融机构，搭建更多更有效的资本平台。积极支持技术创新平台建设。努力争取国家、省支持，争取优势创新资源在张家口布局。支持建立重点实验室、技术创新中心、产业技术研究院等。新建院士工作站3家以上，省级以上研究创新平台5家以上。

（五）促进城乡融合发展。以城带乡，以乡促城，大力实施乡村振兴战略，完善城市功能，提升城市承载力。

加快构建绿色城镇体系。完成市级国土空间规划编制，深化细化各专项规划。完善“五级两规一导则”规划建设体系。加强规划引领和刚性约束，规划即法，执法如山，坚决打击违规建设行为。市、县两级继续实施城市“双修”、基础设施改善、市容提升、老旧小区改造、棚户区改造、路网畅通六大行动。各县区结合易地扶贫搬迁、“空心村”整治和全域旅游，在坚持生态优先的前提下，建设一批中心镇、中心村。全面启动924个“空心村”治理工程。积极推进旅游铁路规划建设。加快立体综合交通体系建设。宁远机场改扩建完成，京张、崇礼、张大、张呼高铁建成通车。延崇高速、张石高速蔚县支线具备通车条件。张尚高速、京藏高速与京新高速连接线开工建设。谋划北环与西环连接工程。年内全市常住人口城镇化率达到58.5%。

扎实推进乡村振兴。实施农业供给侧结构性改革三年行动计划。加快发展现代都市农业、特色高效农业、精品农业，促进奶业振兴。大力推进农业龙头企业提质扩模带行业行动，发展休闲农业、乡村旅游、农村电商等新产业新业态，促进农村一二三产业融合发展。怀来葡萄文化产业园要提升规划，精细推进，在三产融合、协同创新方面起到示范作用，带动怀涿盆地葡萄产业发展整体上档升级。完善农村承包地“三权分置”，稳慎推进农村宅基地制度改革，深化农村集体产权制度改革。积极发展家庭农场、家庭牧场、专业合作社等农村新型经营主体，培育新型职业农民，培养一批农业职业经理人、乡村工匠、文化能人。强力推进农村人居环境整治三年行动，突出抓好垃圾污水处理、“厕所革命”、村容村貌提升。注重宣传组织群众，完善乡村治理机制，建设平安乡村、文明乡村。

全面推进文明城市创建。通过全国文明城市创建促进市民素质和城市品质双提升。深入推进城管综合执法体制改革，严格城管责任体系，实施无缝隙、全天候精细管理。加快出租车、公交、商贸、餐饮、住宿等窗口行业文明提升。大力发展公共交通，强化交通管理，努力缓解交通拥堵、停车难等问题。深入开展“迎冬奥、讲文明、树新风”活动，全市人民团结起来、行动起来，手拉手共创文明城，意气风发、精神抖擞地迎接“精彩、非凡、卓越”的冬奥盛会。

（六）全面推进改革开放。强化服务意识，坚持市场化方向，锐意改革、勇于担当，主动作为、持续用力，激发市场活力。

强力推进“双创双服”优化发展环境。深化“放管服”改革，加快“一网、一门、一次”改革，加快“网上政府、身边政府”建设，编制“马上办、网上办、就近办、一次办”审批服务事项目录，完善“一件事”办事清单和“最多跑一次”事项清单。配强专职办事队伍，重点项目主动上门帮办。整合部门行业数据资源，实现信息共享、一网贯通。实施企业开办提速、证照分离等专项行动，大幅压缩建设项目审批时间。完成全市22条政务服务热线整合升级，打造为民服务的“高效热线”“智慧热线”。“市民中心”6月份挂牌运行。推进“多证合一”扩容升级。以“双随机、一公开”监管为基本手段，以重点监管为补充，以信用监管为基础，构建新型监管体制。深化部门、金融机构、干部包联制度，把“双创双服”包联范围扩展到全部规上企业、限上企业，尽力覆盖中小微企业，随时了解企业困难和诉求，一企一策为企业送“政策包”“服务包”。

强力推进能源革命。着力先进性、示范性。在重点突破上下功夫。全面推进可再生能源示范区 “三大创新”“四大工程”“五大功能区”建设，构建“发储输用”一体化绿色能源体系。努力发挥可再生能源在脱贫攻坚中的重要作用。完善城乡电网规划，加快电网建设。拓展“四方协作”机制应用范围，加强配套支持政策，大力推进可再生能源在“以电替煤”、“以电替油”、大数据和其它绿色产业领域的消纳应用。抓紧推进“低碳奥运专区”“中国数坝”和“氢能利用示范城市”建设。加快±500千伏多端柔性直流和张北至雄安新区1000千伏特高压建设。2019年，全市可再生能源消费量占终端能源消费比例达到26%以上。

强力推进重点领域改革。深化国有企业改革，大力推动混合所有制改革，稳妥推进市级经营性国有资产集中统一监管改革，组建国有资本投资运营公司，抓好职业经理人试点。深化财税金融体制改革，全面实施预算绩效管理。制定有效措施，提高金融服务“三农”、支持实体经济、支持中小微企业的能力，支持企业挂牌上市。加强金融和类金融机构监管，防范金融风险，打击非法集资、非法放贷和金融诈骗行为。深化科技体制改革，创新评价制度，加强知识产权保护，扩大知识产权质押融资，用好“双创”专项金融债券、孵化专项债券和创新券。加快公立医院综合改革，大力推进医联体建设，加强疫苗监管制度建设，推动基本医疗保险支付方式改革，努力便民惠民。

强力推进对外开放。主动融入“一带一路”建设，着力深化国际交往。发展一批友好城市，鼓励园区、企业加强国际合作。落实出口退税政策，壮大外贸出口基地和龙头企业，培育自主出口品牌，提高国际竞争力，提升张家口经济外向度。加快提升开发区能级水平，利用好中国国际进口博览会、高交会、“5·18”经洽会等国家、省重要商务平台，精心打造“零度以下经济论坛”、旅发大会等本地商务平台。全力筹办好“崇礼论坛”。

（七）努力保障改善民生。强化以人民为中心的发展思想，抓重点、补短板、强弱项，加快发展社会事业，使发展成果让人民共享。

大力发展教育事业。把握协同发展、新型城镇化和乡村振兴趋势，依据城乡规划，深化教育专项规划，优化教育资源布局。加大教育投入，加强各级各类学校建设和教师队伍建设。实施学前教育普及工程，55所普惠制幼儿园建成投用。加强乡村小规模学校和乡镇寄宿制学校以及城乡结合部、城区中小学标准化建设，加快市县城区教育资源增量扩容提质，着力解决“大班额”等问题。实施高中教育普及攻坚行动，打造5所特色高中。大力发展职业教育，加强职业教育国际合作，深化产教融合、校企合作，鼓励企业集团兴办职业教育。加快张家口学院新校区建设，积极支持高等教育发展。加强对校外教育培训机构的监管，对无证幼儿园、在职教师有偿补课等开展专项治理，进一步规范教育秩序。

贯彻实施“健康中国”行动计划。加快基层医疗机构标准化建设，推进一批乡镇卫生院与村卫生室一体化管理试点和医联体、医共体建设。推行分级诊疗和家庭医生签约服务，让群众感到医疗服务在身边。实施“提质强基”工程，引进高层次人才、提升医疗水平，招录全科医生、保障基本医疗。启动洋河新区综合医疗中心建设，市第一医院和建国医院完成整合重组，加快提升综合医疗水平。切实加强基本公共卫生服务，做好重大疾病防控工作，推进医养结合型养老服务。

繁荣文化体育事业。加强文化遗产保护利用，推动长城、张家口堡、泥河湾遗址群、蔚县古堡等重点文物保护利用提档升级。抓紧“万里茶道”张家口段申遗和太子城遗址公园规划建设。持续开展文化惠民工程。围绕庆祝新中国成立70周年推出一批精品文艺创作，开展系列文化交流活动，图书馆、博物馆正式开放。积极发展全民健身运动，推进群众体育“六边”工程，高水平举办足球、篮球、乒乓球等6项城市联赛，打造“全季·全体育”品牌。举办第九届市民运动会。

完善加强住房保障和供应体系建设。坚持以政府为主提供基本保障，以市场为主满足多层次需求，推行多主体供给、多渠道保障、租购并举的住房制度。加大公租房、人才房、平价房建设保障力度。新开工棚户区改造13000套，改造老旧小区54个，完成农村危房改造9754户。大力支持农村互助幸福院建设。

强化兜底保障。落实就业优先政策，切实做好各类毕业生、农民工、退役军人、下岗转岗职工等群体就业。加强就业援助，扶持城镇困难人员、残疾人就业，确保零就业家庭动态清零。城镇新增就业6万人以上。深入推进全民参保计划，深化企业养老保险制度改革。完善统一的城乡居民基本医疗保险和大病保险制度，重视关爱特困人员、残疾人、优抚对象等群体，持续提高城乡低保标准。为10485名贫困残疾人提供基本康复服务，为8000名贫困残疾人适配基本型辅助器具，为1550名贫困残疾人提供托养服务补贴。

切实加强社会治理。深化危化品、矿山、道路交通、建筑施工、油气燃气、人员密集场所等重点行业、重点区域、重点部位安全生产专项整治，坚决遏制重特大事故发生。加强应急指挥平台建设，整合应急救援力量，加强基础建设，提高应急能力，加强宣传教育，提高全社会防范安全风险和防灾减灾意识。加强食品药品监管，开展药品、保健品非法营销专项整治。加强和创新社会治安综合治理，坚持发展新时代 “枫桥经验”，抓好矛盾纠纷多元化解、城乡社区网格化服务管理、综治信息化建设，努力打造共建共治共享的社会治理格局。加强组织领导，加强社会动员，深入推进扫黑除恶专项斗争，建设平安张家口、和谐冬奥城。

同时，要大力加强国防教育和国防后备力量建设。大力配合支持部队推进新时代强军事业，切实解决好军转干部安置、随军家属就业和子女入学等问题，加强“两中心两站”建设，做好军队退役人员安置和服务工作。充分发挥工会、共青团、妇联等人民团体桥梁纽带作用。认真做好民族宗教、新闻出版广电、外事侨务、人民防空、史志档案、气象地震、援疆援藏、妇女儿童、老龄等工作。

（八）全面加强政府自身建设。新时代需要新担当，新征程要有新作为。我们必须全神贯注、脚踏实地，努力建设学习政府、法治政府、务实政府、效率政府、廉洁政府，忠诚履职尽责，不辜负党和人民期望。

要旗帜鲜明讲政治。牢固树立“四个意识”，坚定“四个自信”，坚决做到“两个维护”。以党的旗帜为旗帜，以党的方向为方向，以党的意志为意志，在思想上政治上行动上自觉与以习近平同志为核心的党中央保持高度一致，对党忠诚，坚决贯彻党中央决策部署。

要勤奋学习苦钻研。大兴学习研究之风。用习近平新时代中国特色社会主义思想武装头脑、指导实践、推动工作。牢固树立立体思维、辩证思维、逆向思维，深谋细研各项工作，把创新、协调、绿色、开放、共享新发展理念贯彻到政府工作的方方面面，贯彻到每项工作的全过程。

要依法行政善担当。坚决秉承宪法精神，遵守各项法律法规。自觉接受人大法律监督、工作监督和政协民主监督，自觉接受人民群众监督和社会舆论监督，让权力在阳光下运行。

要锤炼作风抓落实。自觉践行“三严三实”，不避事、不畏难、不推拖，向形式主义、官僚主义亮剑，对推诿扯皮现象开刀，同歪风邪气斗争。发扬钉钉子精神，各项工作都要建台账、拉清单、明责任、强督查、求结果，实打实、硬碰硬，抓铁有痕、踏石留印，推动各项工作落地见效。

要清正廉洁树公信。大力加强政府系统党的建设，严格遵守中央八项规定及实施细则精神，深入推进党风廉政建设和反腐败斗争，持续用力正风肃纪，“一岗双责”示范带动，努力打造对党忠诚、热爱人民、廉洁奉公的公务员队伍。要为干事者撑腰、为担当者担当，让襟怀坦白、一心为公的好干部消除后顾之忧，心无旁骛地放手工作。

各位代表，张家口正处在实现创新发展、高质量发展、绿色发展的关键时期。“船到中流浪更急，人到半山路更陡”。“我们都在努力奔跑，我们都是追梦人。”让我们更加紧密地团结在以习近平同志为核心的党中央周围，高举习近平新时代中国特色社会主义思想伟大旗帜，在省委、省政府和市委的坚强领导下，坚定信心，振奋精神，携手并肩，拼搏向上，以斗争精神狠抓各项工作落实，为扎扎实实完成三大历史任务、圆满交出两份优异答卷不懈奋斗！
